# Decoding viewer emotions in video ads

**DOI:** 10.1038/s41598-024-76968-9

**Published:** 2024-11-02

**Authors:** Alexey Antonov, Shravan Sampath Kumar, Jiefei Wei, William Headley, Orlando Wood, Giovanni Montana

**Affiliations:** 1 Department of Statistics, University of Warwick, Coventry, UK; 2 WMG, University of Warwick, Coventry, UK; 3 Alan Turing Institute, London, UK; 4 System1 Group PLC, London, UK

**Keywords:** Emotion prediction, Video analytics, Deep learning, Computer science, Statistics

## Abstract

Understanding and predicting viewers’ emotional responses to videos has emerged as a pivotal challenge due to its multifaceted applications in video indexing, summarization, personalized content recommendation, and effective advertisement design. A major roadblock in this domain has been the lack of expansive datasets with videos paired with viewer-reported emotional annotations. We address this challenge by employing a deep learning methodology trained on a dataset derived from the application of System1’s proprietary methodologies on over 30,000 real video advertisements, each annotated by an average of 75 viewers. This equates to over 2.3 million emotional annotations across eight distinct categories: anger, contempt, disgust, fear, happiness, sadness, surprise, and neutral, coupled with the temporal onset of these emotions. Leveraging 5-second video clips, our approach aims to capture pronounced emotional responses. Our convolutional neural network, which integrates both video and audio data, predicts salient 5-second emotional clips with an average balanced accuracy of 43.6%, and shows particularly high performance for detecting happiness (55.8%) and sadness (60.2%). When applied to full advertisements, our model achieves a strong average AUC of 75% in determining emotional undertones. To facilitate further research, our trained networks are freely available upon request for research purposes. This work not only overcomes previous data limitations but also provides an accurate deep learning solution for video emotion understanding.

## Introduction

Understanding and predicting viewers’ emotional responses to videos is a pivotal challenge due to the inherent complexities of human emotions and the technical difficulties in capturing these nuances from video content. Emotions, profoundly shaping human experience, are influenced by complex cultural norms, psychological states, and physiological responses. Their diverse expressions heavily depend on subtle social contexts and individual differences, presenting significant challenges in accurately deciphering their nuanced complexities. Overcoming these challenges is crucial, given the vast potential applications in video indexing, advertising, and content recommendation, enabling transformative applications in advertising and marketing through granular insight into audience emotional resonance.

Most prior emotion recognition has required direct human observation and annotation, introducing subjectivity and bias. Our study explores a different perspective - predicting emotions purely from multimedia content, without any human input. We develop a deep learning system that annotates videos by hypothesizing the viewer emotions they may elicit, based solely on analyzing the audiovisual signals, devoid of direct human cues^1^. This data-driven approach opens promising opportunities to understand emotions evoked by media in a more objective, scalable way. Potential applications are far-reaching, from enhancing recommendation engines to demystifying psychological aspects of filmmaking. Particularly promising is advertising, where gauging emotional resonance could transform campaign strategies and creative testing.

The examination of emotions in academia is primarily anchored in two key models. The categorical model, founded on Ekman’s pioneering work, asserts that emotions are distinct entities defined by specific expressive and physiological markers^2^. Through extensive cross-cultural studies, Ekman emphasized the universal nature of certain emotional expressions across diverse populations. This provides a robust foundation for analysis and wide-reaching applications like ours. Subsequent research has further refined categorical models, introducing more nuanced labels. Alternatively, the continuous model, exemplified by the circumplex of affect, envisions emotions on a fluid plane defined by valence and arousal^3^. This flexible perspective celebrates the intricacies and relationships between emotions. While the continuous view offers valuable insights into the subtle nature of emotions, we align here with Ekman’s categorical approach for its structure and systematic framework. Clear distinctions between discrete emotions facilitate more straightforward mapping and prediction, streamlining the training process for machine learning.

Our study builds on extensive prior research, leveraging a proprietary dataset from System1 Group PLC, a pioneering leader in advertising effectiveness prediction. This dataset derived from the application of System1’s proprietary methodologies on over 30,000 real video advertisements. Each video was annotated by approximately 75 diverse viewers who provided real-time emotional annotations. This equates to over 2.3 million rich labels across eight categories, enabling us to track nuanced temporal emotion shifts often hidden in traditional feedback. To establish a strong baseline classifier, we develop a tailored convolutional neural network that ingests video and audio to predict evoked emotions in short clips. This model achieves a mean balanced accuracy of 43.6%, with strong performance for detecting happiness (55.8%) and sadness (60.2%). When tested on full-length videos, we obtain an average AUC of 75%, demonstrating robustness despite the inherent subjectivity of emotion.

Despite this initial progress, substantial challenges remain in collecting diverse and unbiased emotional data, and developing algorithms that can learn conceptual connections between raw multimedia and complex psychological states. As part of this study, we are contributing a new dataset consisting of 26,637 labeled 5-second video clips capturing core viewer emotional responses. These clips have been used to produce the results presented in this work and are made publicly available for research purposes, along with our open-source code and trained models. By contributing our processed dataset, code, and model weights, we aim to spur community efforts to advance emotion understanding in video analytics and empathetic AI systems.

## Methods

### Dataset

In our study, we made use of dataset from System1’s Test Your Ad, which was carefully curated by System1 Group PLC. This dataset comprises 30,751 publicly available video advertisements representing various commercial products. These ads were aired between 2017 and 2020 and targeted audiences in both the UK and US. What makes this dataset valuable is that the ads adhere to industry-standard duration, often set at intervals of 5, 15, 30, or 60 seconds. A breakdown of duration times can be found in Table 1.

The annotation process for these video ads was semi-automated and involved a panel of human reviewers sourced from Toluna’s extensive global panel, which is an online platform with over 79 million registered members worldwide for facilitating surveys and research studies. For our study, participants were selected from the UK or the US, matching the country-specific content of the videos, with demographic quotas applied to ensure broad national representation. They were invited to surveys without prior knowledge of the test’s nature, ensuring unbiased responses.

**Fig. 1:**
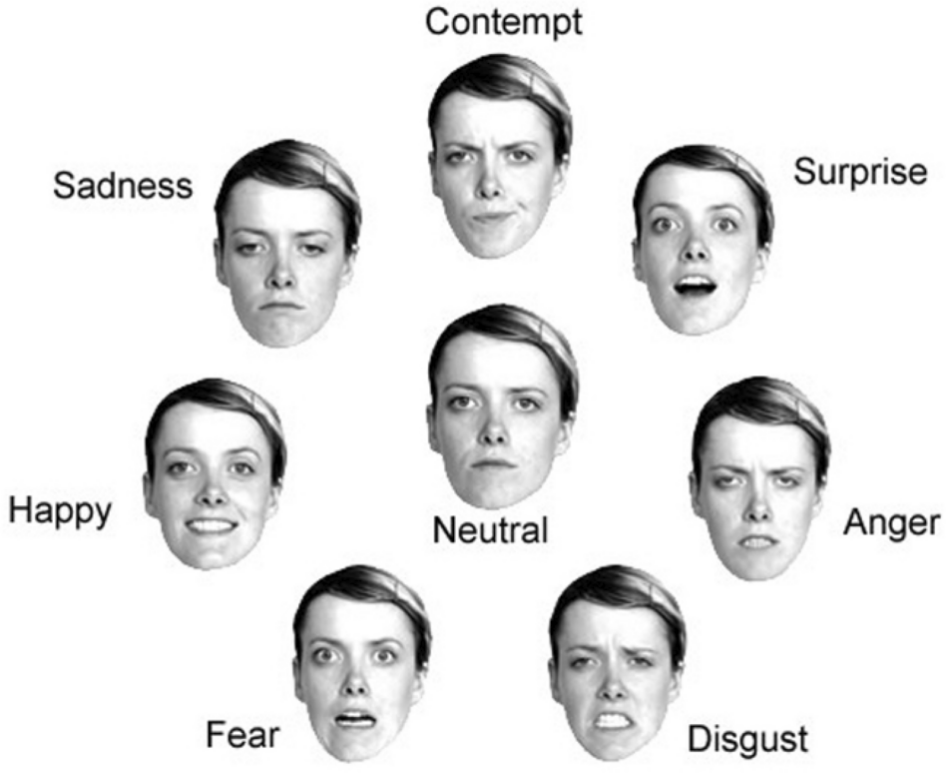
Facial expressions used by System1 Group PLC’s FaceTrace method during the video annotation process. (Source: System1 Group PLC, reproduced with permission).

An intuitive interface called FaceTrace was used, which continuously displayed a range of facial expressions representing different emotions. Viewers could pause the video at any point and select the facial expression image that best matched the emotion they were feeling in that moment. This real-time interface enabled viewers to effortlessly connect their immediate emotional reactions to the corresponding facial expression without having to recall their emotions after watching the entire video. Figure 1 shows the eight facial expressions used in the interface, encompassing Ekman’s seven foundational emotions^2^: anger, contempt, disgust, fear, happiness, sadness, and surprise, as well as a neutral expression. The real-time annotation approach resulted in a sequence of clicks for each video and viewer, each linked to a specific time point and associated emotion. We refer to this series of temporal annotations as the *emotional profile*.

The labeling process involved around 75 participants per video, amassing a total of 2.3 million emotional profiles. Upon closer examination, we found that there were 1.6 million unique completed reviews. In $99.8\%$ of cases, an individual reviewer assessed only one or two distinct video advertisements. While individuals may have subjective emotional reactions influenced by personal biases and experiences, consolidating annotations from 75 participants per video allowed us to distill a more robust and objective representation of the emotional impact evoked by the video content, transcending the limitations of individual, subjective signals.

### Quantification of ground-truth emotional responses

In our endeavor to trace the nuanced shifts in viewers’ emotional experiences while viewing videos, we introduce the term *emotional jump*. This term captures a quantifiable shift in emotional intensity that viewers undergo during a brief segment of a video. Depending on the content and viewer interpretation, the intensity of this jump might either escalate or diminish. Our methodology comprises two phases. Initially, we identify patterns of these emotional jumps across all the brief segments within our dataset. Subsequently, we categorize each jump in accordance with these established patterns.

To elucidate further, let us define some notation. Let $s_t^e$ be the share of viewers experiencing emotion $e$ at time $t$; $b_t^{e,i}$ is a binary marker for the viewer $i$ showing if they felt emotion $e$ at time $t$ (1 for yes, 0 for no), and $n$ is the total viewer count for a video. The *strength of emotion* $e$ at a given time point $t$ is:

1 $$\begin{aligned} s_t^e = \frac{\sum _{i=1}^n b_t^{e,i}}{n} \end{aligned}$$

**Fig. 2:**
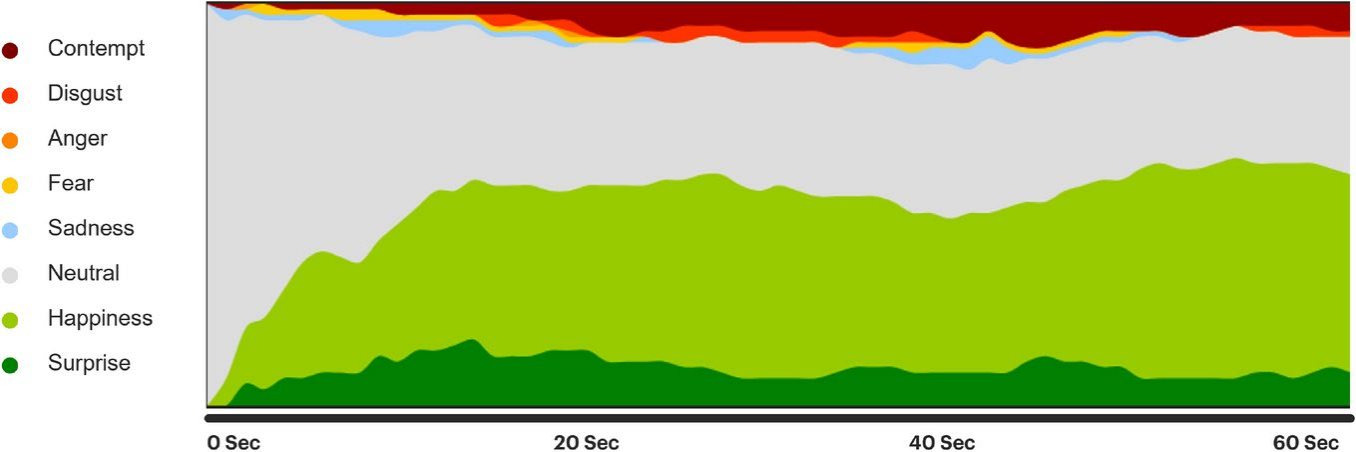
System1’s Test Your Ad: At each time point throughout a video clip, we can measure the proportion of viewers in the panel (approximately $n=75$) who self-declared experiencing one of the eight emotions. This example illustrates the changes in emotional profiles within a video.

Figure 2 provides a graphical representation, illustrating the fluctuations in the strength of emotional signals over time based on the content of a specific advertisement.

Our methodology predominantly focuses on analyzing short video segments, specifically segments that are 5-seconds in length. This concise duration facilitates the capture of rapid emotional transitions, which might otherwise be diluted in longer segments. Additionally, the granularity of these segments allows us to correlate emotional shifts with specific moments in the video, shedding light on the most evocative sections. To dissect the video into these segments, we employ a sliding window approach, advancing one second at a time, thereby examining all possible 5-second combinations within a video. The difference in emotional intensity at the beginning and end of each segment represents the *emotional jump*,

2 $$\begin{aligned} J^e = s_{start}^e - s_{end}^e. \end{aligned}$$

If $J^e$ is positive, it means viewers felt more of that emotion by the end of the clip. Upon extracting emotional jumps from each video, our pivotal task is to identify which of these signify a *noteworthy* emotional transition. For each emotion, we analyze its corresponding jumps within the empirical frequency distributions. If a given $J^e$ resides within the top $k$th quantile of its associated emotion’s distribution, it is assigned a corresponding label, highlighting a significant emotional jump.

Transitioning from continuous to discrete signals was a considered decision, driven in large part by the inherent challenges of continuous emotional metrics. These signals, despite their granularity, often contain noise from multiple sources. For one, viewers might not uniformly respond to the same video stimuli at identical times, leading to variances in emotional trajectories. Additionally, sporadic viewer reactions might not necessarily correlate with the overarching sentiment of the video. Such noise complicates subsequent analysis, obscuring genuine viewer sentiment. In contrast, discrete labels offer a distilled representation of emotions, mitigating ambiguity and creating clear boundaries between distinct emotional states. By basing our analysis on the full distribution of emotional jumps, we can more authentically capture viewer sentiment, avoiding fleeting or anomalous blips. Moreover, discrete labels align smoothly with machine learning frameworks tailored for classification tasks, simplifying both modeling and interpretation. By setting a threshold at the upper *k*th quantile, our methodology focuses on labeling only the most pronounced emotional shifts as positive examples. This makes the model more robust against noise and improves generalizability by ensuring it learns to recognize strongly evident emotional cues. A higher labeling threshold yields cleaner, better-defined training data consisting of salient examples, rather than attempting to fit indistinct or borderline cases.

### Emotional jump classification

Given a dataset $\mathscr {D} = \{ v_i, y_i\}_{i=1}^N$ of *N* 5-second video clips $v_i$, each labeled with one of 8 discrete emotions $y_i$, our primary goal is to predict the conditional probability distribution *p*(*y*|*v*) for an input clip *v*. Formally, we want to learn a function $p = f(v)$ that outputs an 8-dimensional probability vector *p* given *v*. The complex spatio-temporal patterns in this visual classification task warrant a computer vision model capable of capturing nuanced features.

Convolutional neural networks (CNNs) are well-suited due to their hierarchical representations and exceptional ability to extract visual features, as evidenced across diverse applications^4–6^. Specifically, we employ the Temporal Shift Module (TSM)^7^ which leverages 2D CNNs with frame shifting to achieve computational efficiency without compromising representational power for video tasks like action recognition. This aligns with our insight that video-evoked emotions depend on both immediate frames and preceding context. However, in its original form, TSM lacks integration of audio, which often serves as an emotional amplifier. To address this, we enhance TSM by converting audio to mel-spectrogram representations and adding them as input frames. Mel-spectrograms capture perceptual properties of human hearing for intuitive audio features. This multimodal architecture, termed TSAM, synergistically fuses visual and auditory modalities.

**Fig. 3:**
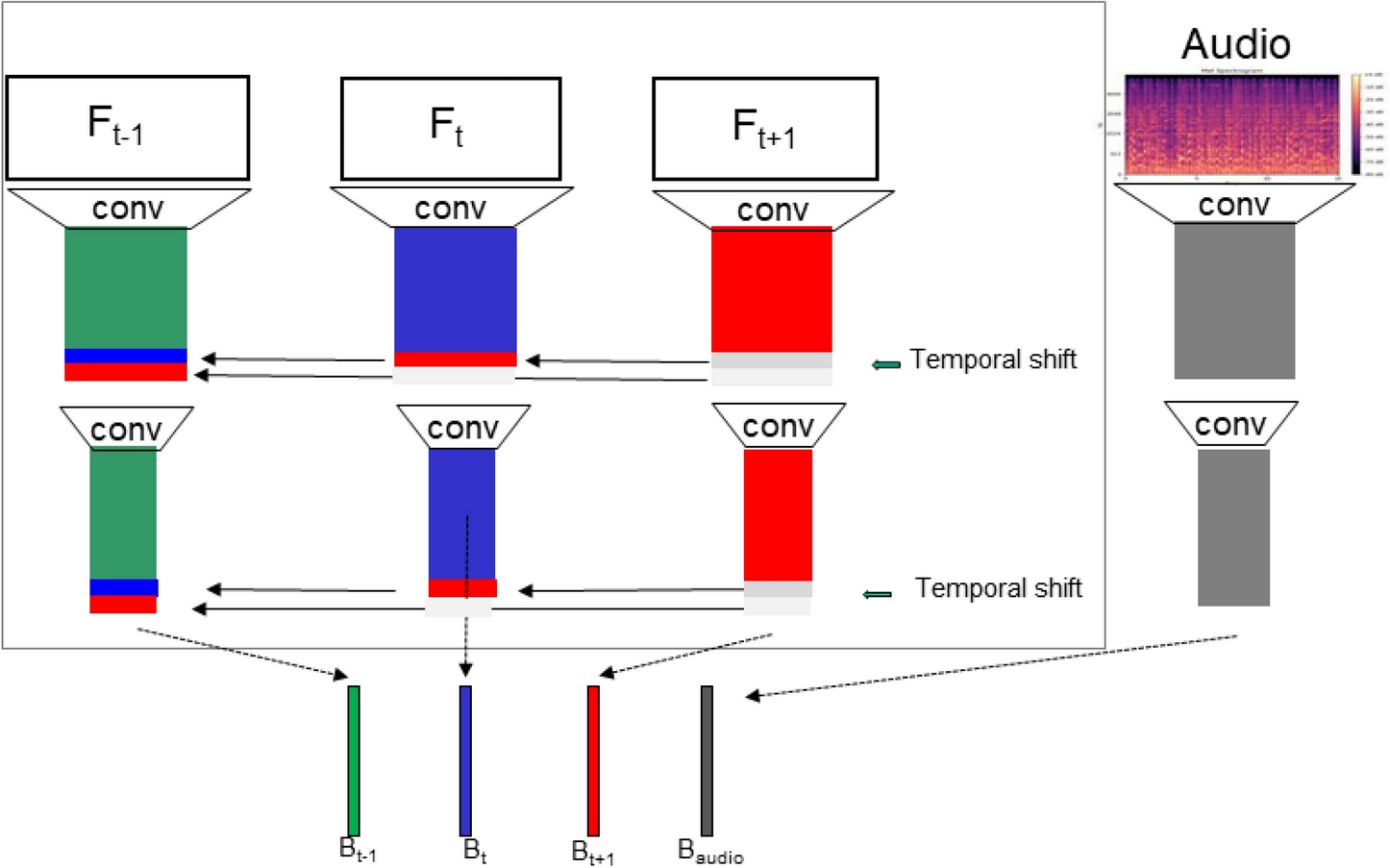
TSAM model: the multi-modal CNN architecture takes as input a predefined number of video frames (video segments) and audio converted into mel-spectrograms (audio segments). The ResNet50 backbone is used to extract features from both video and audio segments. Features from video segments are shifted between each other at different blocks of ResNet50. The audio input is represented by the mel-spectrogram) and is processed by the same backbone without shifting. The extracted features are fused by averaging and mapped to the output classes using a fully connected layer.

In our TSAM architecture, we utilize ResNet50^8^ as the backbone to jointly process video frames *v* and audio mel-spectrograms *a*. While *v* undergoes temporal shifting per TSM, *a* bypasses this. Both modalities pass through the same ResNet50 weights, enabling shared spatio-temporal feature extraction. Post-processing dropout is applied to mitigate overfitting. We then aggregate the visual and auditory representations by averaging their features. Finally, a fully-connected layer generates predictions $\hat{y}$ for the 8 emotion classes based on this multimodal embedding. We train end-to-end by minimizing the cross-entropy loss $L(\hat{y}, y)$ between predictions $\hat{y}$ and ground truth labels *y*. Figure 3 presents a schematic of this architecture. The implementation of the TSAM architecture has been released as an open-source project (https://github.com/aav-antonov/TSAM).

For training, validation, and testing, we sample a fixed number of frames *k* from each input video *v* using uniform sampling. Specifically, we divide *v* temporally into *k* equal segments, then randomly select one frame per segment. This generates a representative subset of *k* frames covering the full *v*. Our frame dataset consists of $256 \times W$ resolution images, with variable *W* based on original video aspect ratios. Keeping *k* consistent enables fair evaluation across diverse videos. The 256 pixel height allows efficient batch processing on GPUs. Overall, this uniform subsampling extracts informative training frames from *v*.

Our decision to use random frame selection within equally divided segments of each 5-second window was primarily driven by the goal of balancing computational efficiency with capturing diverse emotional expressions across the large video corpus. This approach aimed to mitigate potential biases and overfitting that could result from consistently choosing frames based on predefined criteria, which may not generalize well across the wide range of video content and emotional contexts in our dataset. Furthermore, this randomized approach allowed us to explore the temporal dynamics of emotional expressions in a way that is computationally feasible and reflective of the natural variability and complexity of human emotional responses to video advertisements. While there exist more sophisticated frame selection techniques, such as those based on peak emotional expression detection or content saliency, we plan to investigate and evaluate these alternative methods in future work to further refine our approach and contribute valuable insights to the field of video-based emotion recognition.

During training, we use data augmentation including random $224 \times 224$ cropping and horizontal flipping to improve robustness. For validation and testing, we use centralized $224 \times 224$ crops to standardize inputs. We optimize hyperparameters like dropout, batch size, learning rates, and epochs using the validation accuracy. The best performing model on the validation set is selected as the final model for testing. This augmentation and tuning procedure enhances diversity and reduces overfitting, resulting in optimal model generalization.

### Full-length video analysis using sliding windows

Thus far, we have utilized a convolutional neural network (CNN) trained on 5-second video clips to classify emotions. However, comprehensively analyzing video advertisements requires modeling emotional responses evoked by full-length videos. Directly applying our clip-trained CNN poses challenges. Emotions manifest non-uniformly in longer videos, with certain segments eliciting intense yet transient responses while other periods remain neutral. Brief emotional peaks may become obscured when analyzing full videos holistically. Furthermore, temporal dynamics and context between segments provide important emotional insights.

To enable nuanced modeling of emotion responses to full video ads, we propose a sliding window technique. This approach synergistically combines fine-grained localized analysis of segments with consolidated scores summarizing dynamics across the full video duration. Specifically, it involves extracting overlapping 5-second segments across the video, progressing in 1-second intervals between segments. For a 30-second video, this process yields 26 overlapping windows spanning its entire length. Subsequently, each segment is classified independently by our TSAM, producing an 8-dimensional probability vector indicating the likelihood of each emotion. If an emotion’s probability exceeds 0.5 for a given segment, that emotion is designated as present. After assessing all windows, we consolidate the counts $C^e$ for each emotion and normalize by the video duration as $S^e = \frac{C^e}{T-4}$ where T is duration and T-4 is the window count. An emotion is deemed present if its score meets threshold $t_C$.

This multifaceted approach has several motivations. Video ad emotions often manifest non-uniformly, with transient yet intense bursts in certain segments. Analyzing overlapping windows helps capture even brief peaks, retaining nuance. Furthermore, we want an expansive perspective of the video’s overall emotions, not just isolated segments. This consolidated view allows holistic analysis to balance detecting the overarching tone and local peaks.

## Results

### Descriptive statistics of viewer clicks and emotional jumps

**Fig. 4:**
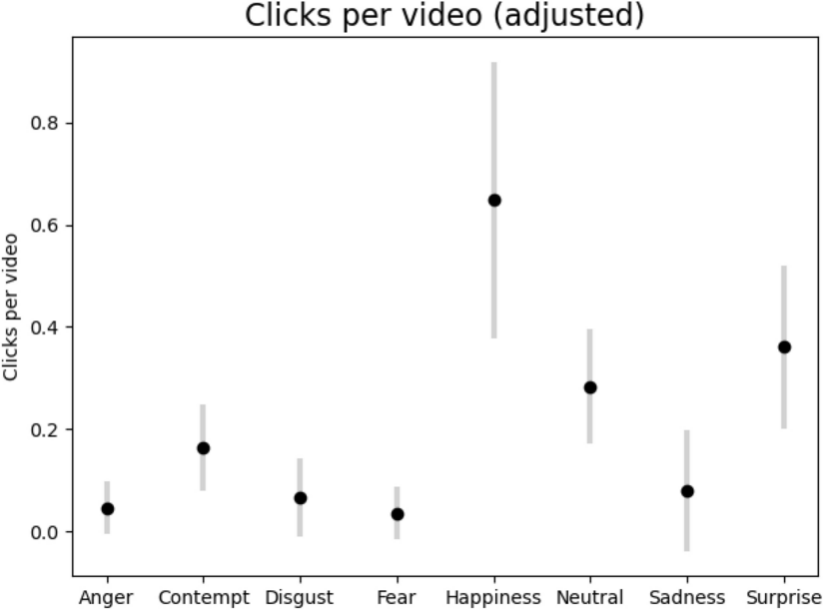
System1’s Test Your Ad data: Average number of user clicks per emotion for every 30 seconds of video, adjusted to account for different video lengths.

To begin our analysis, we first examine the distribution of video lengths present in our dataset. Table 1 shows the distribution of video ads by duration, in seconds. As illustrated, our dataset is composed primarily of 30-second commercials, followed by 15-second and 60-second ads. Figure 6 depicts the distribution of number of clicks per user for each of the eight emotions, without normalizing for differences in video duration. When we normalize for a standard 30-second video length and look at the average number of clicks per user in Figure 4, happiness, surprise, and sadness emerge as the most frequently expressed emotions. Overall, our dataset provides a rich portrait of emotional responses, with happiness, surprise and sadness being the predominant emotions expressed. Although viewers could indicate “no emotion,” the data reveals they did not often return to a neutral state after reporting an initial emotion. On average, users expressed 1.4 emotions per 30-second clip, while selecting “no emotion” only 0.3 times.

**Fig. 5:**
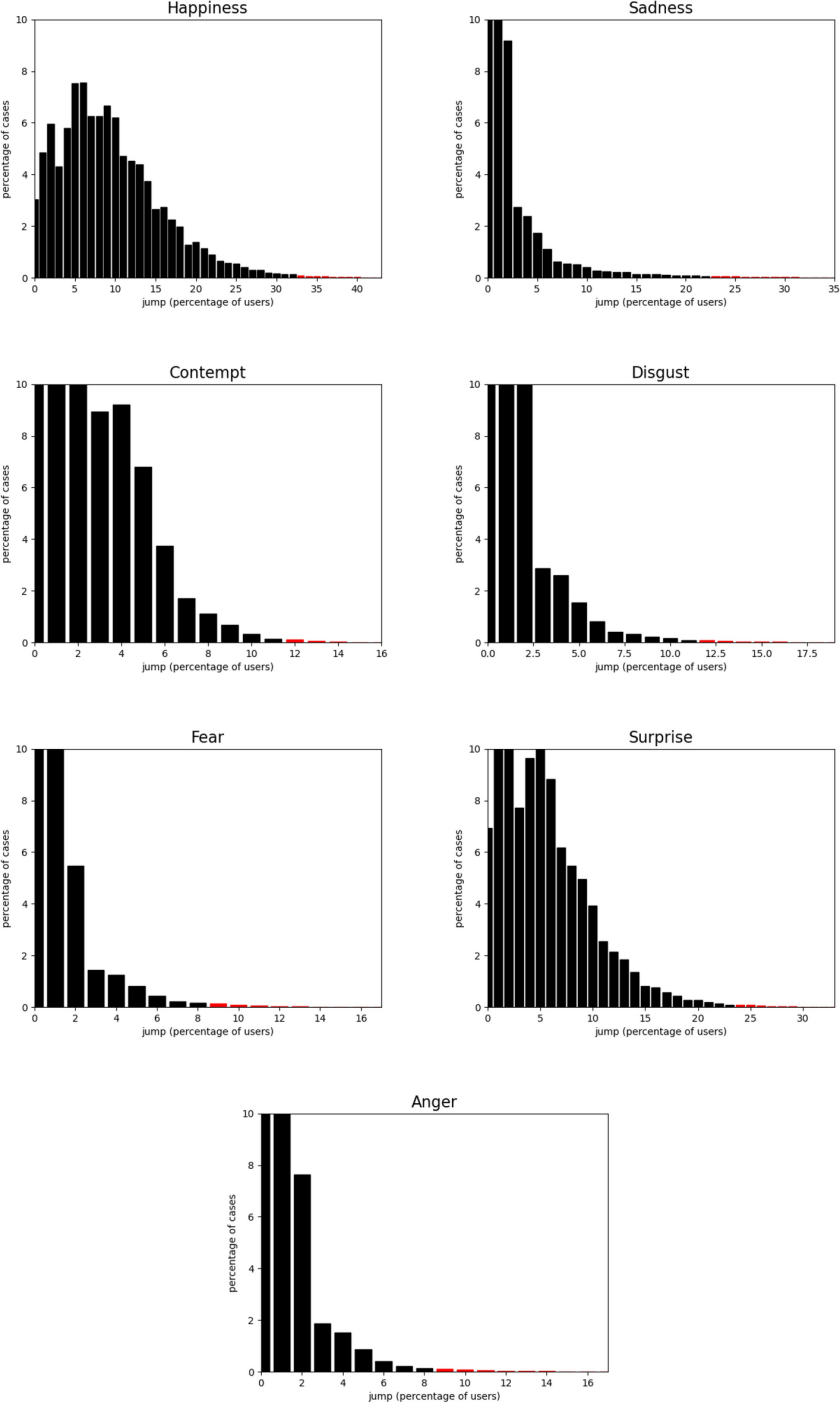
System1’ Test Your Ad dataset: Distribution of 5-second clips based on the percentage of users expressing various emotions. The x-axis shows the response strength as the percentage of viewers feeling the emotion. The y-axis shows the percentage of clips evoking that response level. Clips in the top 0.5% of the distribution (highlighted red) were used to define the emotional jumps, which were labeled for classifier training and testing.

The empirical distribution of 5-second clips based on the percentage of users expressing various emotions is reported in Figure 5. This graph illustrates the intensity of viewers’ emotional responses as a percentage alongside the proportion of video clips that triggered each level of response. Clips in the top 0.5% of the distribution, highlighted in red, were used to define the emotional jumps, which were labeled for classifier training and testing.

By applying a 5-second sliding window to all video clips, we extracted approximately 711,000 labeled segments. Table 2 provides the number of unique 5-second clips inducing an emotional shift for each emotion, along with the total number of distinct full videos containing these 5-second clips. Figure ?? presents sample frames which correspond to those emotional jumps. The purpose of showcasing these frames is to provide examples of what viewers were observing when they selected the respective emotions, offering a visual reference for the emotional peaks recorded.

### Performance of emotional jump classification

For training and testing purposed, we initially split the entire set of 30,751 video ads into three sets of randomsly assigned clips: training (80%), validation (10%), and testing (10%). The classification model was trained and validated on the first two sets, then tested on the held-out testing set. In this section, we report on the classification results and study their dependency on three important design factors: the number of video frames sampled within each clip, the choice of the neural network’s pretraining weights, and the modality of input used for classification, whether solely video or video in conjunction with audio. Our primary metric to assess emotion prediction performance from short clips was the classification accuracy-this metric aligns with conventional norms adopted in tasks like action recognition^7,9–11^.

Drawing from the wealth of prior research on video-based action recognition, it’s known that augmenting the frame samples and harnessing pretrained neural networks, especially with ImageNet weights^12^, generally elevates classification efficacy. This is observed in comparison to limiting the frame samples or initializing weights randomly^7,11^. A noteworthy mention is the ResNet50 network pretrained on ImageNet21K (INET21K), which has garnered acclaim for its performance across a spectrum of benchmark computer vision tasks^13,14^. A comprehensive view of our classification outcomes for the test set can be found in Table 3. The peak performance, a commendable $43.6\%$, was realized with a combination of 16 frames, both video and audio modalities, and a network trained on INET21K weights. Our analyses underscore the importance of a multimodal strategy, as harnessing both video and audio channels demonstrably uplifted the classification performance. Using multimodal inputs led to gains of around 3–4% across configurations compared to unimodal inputs. Interestingly, the benefits of increasing frame counts appeared to plateau—going from 4 to 8 frames provided minor enhancements, while further additions stagnated. Furthermore, the marginal gain from INET21K weights over ImageNet was minimal, only around 0.4%.

A deeper analysis of accuracy by emotion, shown in Table 4, reveals the classifier performed best at identifying sadness, surprise, fear, and happiness. The high performance for detecting these emotions may stem from their common occurrence and expression in the video clips. As these tend to be more universally felt and displayed compared to anger or disgust, for example, the model likely benefits from their stronger representation in the dataset. The abundance of sadness, surprise, fear, and happiness examples enables more robust learning of associated visual and audio cues. Overall, our multimodal approach leverages complementary video and audio signals to improve classification, although frame inputs exhibited diminishing returns. Finally, Supplementary Material Table S2 shows the average classification accuracy obtained on the test set when using different cutoff percentiles of the emotional jump distribution to define positive examples for model training.

### Emotional jumps and *Star rating*

The star rating of a video ad serves as a widely recognized metric for ad quality in the marketing sector. Marketing consultancy agencies often strive to achieve higher star ratings for ads, as they reflect user perceptions and are calculated by averaging users’ emotional responses. System1’s *Star Rating* score ranges from 1 to 5+, where 5+ signifies the highest possible quality and 1 represents the lowest. Five-star ads typically evoke happiness in the audience, leading to an increased likelihood of future commercial success. The System1’s Test Your Ad dataset annotates all videos with star ratings and emotional responses. To underscore the importance of emotion jumps, we highlight a strong correlation between the presence or absence of emotion jumps in an ad and its star rating.

Our findings are detailed in Supplementary Material Table S1, where we divided all videos from the System1’s Test Your Ad dataset into five classes based on their star ratings. The first column of the table displays the distribution of videos within each corresponding star rating class (1+ signifies a rating between 1 and 2, 2+ between 2 and 3, and so on). As observed, 307 ads achieved the highest 5+ star rating, while 15,186 ads were rated 1+. Subsequently, for each class, we calculated the proportion of videos containing at least one emotion jump associated with a specific emotion. The second column presents the respective proportions for happiness jumps. Notably, $48.8\%$ of top-rated video ads (5+) feature at least one happiness-related emotion jump, whereas less than $1\%$ of videos in the 1+ category exhibit a similar characteristic.

### Predicting emotional jumps in full-length video ads

In previous sections, we focused on classifying short video clips for emotional jumps. Now, we extend our efforts to the challenge of predicting these jumps in full-length video ads. Recognizing emotional jumps such as those indicating happiness can serve as a gauge for ad quality and viewer engagement. In our methodology, we first detect emotional jumps per time series clip within each ad using our refined classifier. We then aggregate these detected jumps to enrich our prediction signal-for instance, by counting the total number of jumps per emotion.

Our performance assessment relies on the Area Under the Curve (AUC), a robust measure of the model’s discriminative power between ads with and without specified emotions. Across the test set’s full-length video ads, we observed a significant variance in the model’s performance by emotion. Sadness prediction was notably accurate, with an AUC of 0.88-the highest among the emotions. Happiness and fear also yielded strong AUC scores of 0.78 and 0.83, respectively, underscoring the model’s capability in detecting these emotional changes. Contrastingly, the model’s performance was less impressive for other emotions: contempt and anger both registered AUC scores of 0.67, with disgust slightly above at 0.69. Surprise was identified with moderate accuracy, as reflected by an AUC of 0.73. These results imply that certain emotions, especially those that are subtler like contempt or less defined like neutral-the latter of which is implied to approach the randomness of its detection-pose greater challenges for the model. The corresponding ROC curves for all the emotions can be found in Figure 7.

By averaging the AUC scores across all emotions (excluding neutral), we obtained a mean of 0.75. This average highlights the model’s overall effectiveness in recognizing emotional variations across a broad range of emotions. However, it also points to a potential area for refinement, especially in identifying emotions that are inherently more nuanced or less pronounced in video advertisements.

### TSAM performance on benchmark video datasets

Despite our primary focus on emotional jumps in ads, we conducted a validation exercise by testing our TSAM model on two well-established video classification benchmarks, and specifically for action classification. These public dataset included audio, and served two key purposes. First, it enabled assessing the model’s adaptability to new tasks beyond its original purpose. Second, it highlighted the value of our multimodal approach incorporating audio cues. While not directly related to identifying emotions, performance on these benchmarks was important to verify the model’s performance and versatility before deployment on our primary task.

Specifically, in our experiments, we used two well-known action recognition benchmarks: Kinetics-400 and Something-Something V1. Kinetics-400 consists of 400 human action classes, while Something-Something V1 comprises 174 fine-grained actions. The large number of classes make these challenging benchmarks to evaluate model capabilities. Both focus on classifying directly observable physical motions and object interactions. In contrast, our task of emotion classification deals with inferring the abstract affective state expected to be evoked in viewers. This is substantially more challenging than categorizing visible actions and behaviors. By first validating TSAM on established action datasets, we verified model capabilities on complex tasks with many classes before specializing for nuanced emotion identification, which constitutes a more abstract, complex problem despite fewer output categories.

On Kinetics-400, Supplementary Material Table S3 shows TSAM achieves substantial accuracy gains of  1.5–2% by incorporating audio and  2–2.5% with ImageNet21K weights over ImageNet 1K. These results showcase the benefits of TSAM’s multimodal design and large-scale pretraining. Additionally, Supplementary Materials Table S4 reveals TSAM attained significant performance improvements on Something-Something V1 by incrementally increasing temporal aggregation (raising *k*) from 1 to 4, unlike the limited k in traditional TSM. Remarkably, even without optical flow inputs, RGB-only TSAM ranks among top-performing 2D CNN architectures that utilize motion features. Our results demonstrate competitiveness at recognizing granular actions, providing an important baseline prior to emotion classification. The results also confirm the benefits of our multimodal, temporally-aware approach before specializing for emotion classification.

## Discussion

### Significance of the results

This study tackles the challenge of predicting viewer emotions from video content, an important problem in the realm of effective video advertising. Existing research has emphasized that videos eliciting robust emotional reactions from viewers-whether uplifting or distressing–are approximately twice as likely to be shared and generate higher engagement compared to less emotionally impactful ones^15,16^. Consequently, the ability to determine if a video advertisement will evoke notable emotional responses in viewers is vital for impactful marketing strategies.

To address this problem, we leverage a unique dataset and analytical approach. Our investigation utilizes an annotated database of 30,751 video advertisements, with approximately 75 viewers annotating eight distinct emotions for each video. Unlike existing research that primarily focuses on physiological signals or explicit behavioral indicators, our work emphasizes viewer-reported emotional responses, providing a direct measure of the emotional impact of the video content. We introduce the concept of ’emotional jumps’ - significant shifts in viewer emotional responses identified within brief time intervals of video content. These emotional jumps represent moments where a strong emotional response was evoked across the entire panel of viewers, helping to address issues of subjectivity by capturing a collective emotional resonance. The jumps are crucial for pinpointing moments that profoundly impact viewer emotions, a vital aspect for understanding the emotional effects of multimedia content. Our approach leverages these emotional jumps to build a video classifier.

A key contribution of this work is the development and application of a multimodal convolutional neural network, with the intention of establishing a strong baseline for predicting emotional jumps from video and audio data. This model makes joint use of both modalities and achieves a mean balanced accuracy of 43.6% for 5-second clips featuring pronounced emotional jumps, and an average Area Under the Curve of 75% for full advertisement analyses. These results significantly outperform the random guess baseline of 12.5%, demonstrating the model’s promising ability to distinguish nuanced emotional states within complex video content.

In addressing the classification challenge presented by our dataset, the model exhibited promising accuracy and highlighted the important role of audios signals in emotion identification. Adjustments in frame count or the use of pre-trained weights from INET21K [1, 2] had minimal impact on the outcomes, suggesting a nuanced balance between model complexity and dataset subjectivity. Additionally, our strategic use of a sliding window technique for predicting emotion jumps within complete video ads showed promising efficacy, particularly for emotions pivotal to advertising effectiveness: Sadness, Happiness, and Fear.

Furthermore, we provide an important contribution by making available an unprecedented dataset for research purposes. The dataset consists of a processed subset of System1’s Test Your Ad collection, comprising 26,635 labeled 5-second clips that encapsulate the core of viewer emotional responses to the video advertisements. To the best of our knowledge, this is the first large-scale dataset to provide self-reported, sample-based emotional labels associated with video and audio data. Together with our open-source code and trained models, these resources represent a significant step towards fostering innovation in the field of affective video analysis, enabling researchers to build upon our work and advance the state-of-the-art.

### Connection to other video understanding tasks

Our contributions lie within the domain of automated video understanding, a significant challenge at the intersection of computer vision and artificial intelligence. Video understanding extends traditional image recognition by introducing a temporal dimension, enabling the exploitation of motion and the intricate patterns that unfold over time. Among the multitude of tasks within this domain, human action recognition stands out, involving the identification of various human actions in video frames^9,17^. In comparison to our research, human action recognition focuses on explicit pattern recognition. Actions are observable, tangible, and can be directly inferred from visual content. Extensive efforts from the community have led to the annotation of numerous videos showcasing diverse human actions, resulting in the creation of high-quality, large-scale datasets that serve as benchmarks in the field^18–21^.

Approaching the core of our study, our trajectory intersects with affective video content analysis, a niche area that aims to unravel how videos evoke emotions in humans and predict these emotional responses to dynamic visual stimuli^1,22^. Another tangent within video understanding is facial emotion recognition, involving the detection of human faces in videos and decoding facial expressions to infer underlying emotions^23,24^. It’s essential to note that our study differs in focus. Instead of relying on direct visual cues related to video viewers, we predict emotions evoked by video content itself-a challenge compounded by the fact that emotions, unlike actions, are intangible entities, not directly displayed on video canvases but rather elicited within viewers.

### Choice of neural network architecture

Over the past decade, convolutional neural networks (CNNs) have found success in video understanding, particularly in action recognition^7,10^. Mapping a video sequence to an outcome involves processing both spatial and temporal information. While 3D CNN architectures have been used for video feature extraction^10,25^, they come with a high computational cost. High-performance 3D CNN architectures are often hindered by this computational expense^11^. Various solutions have been proposed to address this issue^10,18^. More recently, transformer-based video networks have shown superior performance on benchmark datasets compared to CNN networks^26,27^. However, transformer-based architectures still come with substantial computational costs and tend to outperform CNNs only when a large amount of data is available^28^.

In contrast, 2D CNNs offer computational efficiency. While 2D CNNs have been used to learn spatial features from individual frames^29,30^, they cannot model temporal information on their own. To overcome this limitation, the Temporal Shift Module (TSM)^7^ was introduced, significantly improving performance on action recognition datasets, even when compared to 3D CNN architectures. TSM employs a ResNet50 architecture to process input frames in parallel and shifts features between temporally neighboring blocks before the convolutional layers. This feature exchange across neighboring frames greatly enhances the architecture’s ability to learn temporal features.

In our work, we leverage recent advancements in this area and extend existing state-of-the-art architectures to suit our problem setting. We chose to adapt TSM for the classification of emotion jumps due to the trade-off between available data, computational efficiency, and classification performance. Furthermore, both visual and auditory stimuli contribute to a video’s affective content^31^. Therefore, we aimed to jointly account for video and audio content when developing our convolutional neural network (CNN) architecture (see the appendix for more details).

### Connections to related studies and datasets

Affective video content analysis exists within a diverse landscape of datasets, but it often lacks depth and breadth when compared to other domains like action recognition, which boasts ample datasets^32^. One notable dataset in this realm is the DEAP dataset^33^, which provides insights into reactions to music videos through both physiological signals and viewer ratings. However, it is limited in scale, containing only 120 one-minute videos. Additionally, other datasets such as the WikiArt Emotions Dataset^34^ and the Discrete LIRIS-ACCEDE dataset^35^ take different approaches, focusing on art emotion annotation through crowdsourcing and affective video annotations using a pairwise comparison protocol, respectively.

Some research in affective video content analysis incorporates external mechanisms like facial expression imaging or physiological measurement devices to discern viewer reactions to videos^36–38^. An example of this approach is the EEV dataset^39^, which utilizes viewers’ facial reactions for automatic emotion annotation in videos, albeit with the challenge of converting facial reactions into distinct emotion labels. Moreover, datasets like the EIM16 dataset, derived from the LIRIS-ACCEDE database, and the Extended COGNIMUSE dataset emphasize different aspects of emotional annotation. The EIM16 dataset, for instance, is designed for both short video excerpt emotion prediction and continuous emotion annotation for longer movie clips, while the COGNIMUSE dataset delves into the distinction between intended and expected emotions.

In comparison to these datasets, our dataset exhibits distinct features. Rooted in manual annotations, it ensures a richer and more personalized emotion spectrum. What sets our collection apart is its remarkable scale, comprising over 30,000 video ads-only the LIRIS-ACCEDE dataset comes close in magnitude. Furthermore, our dataset benefits from the consistency of cultural backgrounds among annotators, offering a unique perspective. This contrasts with datasets like LIRIS-ACCEDE, which source annotations from a global participant pool. The diversity in the emotional spectrum our dataset covers, spanning eight distinct emotions, establishes it as a robust and comprehensive resource for in-depth affective video content analysis.

### Limitations of this study

While our dataset represents an important step forward, we must acknowledge the inherent subjectivity in manual emotion annotation based on viewer recall. Labeling sentiments poses more reproducibility challenges compared to labeling relatively objective actions. However, we can reasonably expect more consistent labeling for pronounced emotional reactions, where a large majority of viewers concur within a short span, as opposed to subtle responses.

To help mitigate subjectivity, we introduced “emotion jumps”—brief clips that trigger particularly intense responses tied to specific emotions. By focusing analysis on these pronounced spikes, we aimed to capture the most vivid and consistent reactions across viewers. We restructured the dataset into a video classification format by selecting clips that provoked heightened emotional reactions for each category. This process yielded improved consistency and reliability in the labels compared to subtle responses. It enabled our model to more effectively discern emotions evoked by videos based on detected patterns in these pronounced responses.

Our approach analyzes full-length videos by extracting overlapping 5-second segments. This sliding window technique aims to capture even brief, transient emotional peaks that non-overlapping segments would likely overlook. Focusing solely on complete videos risks missing these potent yet ephemeral reactions. However, overlapping windows introduce potential drawbacks like redundancy and overemphasis on momentary blips rather than overall sentiment. Ultimately, the sliding window methodology strikes an effective balance. It combines fine-grained localization of emotional bursts with consolidation to assess overall video sentiment. This twin perspective enables nuanced evaluation attuned to the intricacies of emotion dynamics in videos. Alternative methods may neglect critical nuances - either losing transient peaks in holistic views or lacking broader context from non-overlapping segments. Our approach fuses detailed and big picture analysis to provide the layered insight essential for video emotion understanding.

While our study employs a CNN-based model complemented by the Temporal Shift Module to establish a straightforward and computationally tractable baseline, we acknowledge the potential benefits of more advanced architectures, such as those incorporating attention mechanisms or visual transformers. These architectures have demonstrated superior performance in various video analysis tasks, and exploring their application to video emotion recognition presents a promising avenue for future research. Building upon the foundation established in this study, we plan to investigate the potential of attention-based models and visual transformers to further refine our understanding of video-induced emotions, while carefully evaluating the trade-offs between model complexity, computational requirements, and performance gains, considering the unique characteristics of our dataset and task.

### Conclusions

Our prototype represents an original advancement in emotion recognition, leveraging deep learning to predict emotional reactions directly from video content. By analyzing multimedia signals to identify moments that elicit strong responses, our model enables advertisers to create more impactful campaigns that intimately resonate with target audiences. By utilizing our evoked emotion recognition model, marketing professionals can rapidly analyze vast video databases, identifying moments that trigger powerful emotional responses. These emotionally charged segments serve as valuable resources for marketers, enabling them to create compelling and influential advertisements that resonate seamlessly with viewers. The implications are manifold-not only enhancing marketing effectiveness but also fostering deeper and more meaningful engagements with the intended audience. Beyond advertising, applications are far-reaching in luxury brand marketing, entertainment analytics, and empathetic AI systems.

There are numerous avenues for future exploration and improvement. A pressing need exists to elucidate our model’s inner workings and decision-making rationale. As it currently operates as a *black box*, deciphering techniques to highlight precise emotion-evoking frames or elements within those frames would provide valuable insights. Illuminating which visual motifs or ambiance the model associates with certain emotions could allow advertisers to refine strategies for maximal emotional impact. Diversifying the cultural demographics in our dataset is another important area for improvement. While currently spanning the UK and US, expanding to broader global regions could enhance applicability. Emotional triggers and norms differ significantly across cultures. For instance, reactions in Asia or South America may deviate markedly from the current Anglo-centric data. By diversifying cultural representation, our model could learn more universal emotional patterns.

**Table 1:** System1’s Test Your Ad data: distribution of video ads by duration (in seconds).

Duration	Number of Videos
5	229
10	1640
15	8548
20	1811
30	15,123
40	263
45	105
60	2218
75	39
120	297
Other	481

**Table 2:** System1’s Test Your Ad data: Number of labeled 5-second video clips and corresponding number of videos, categorized by emotion.

Emotion	No of clips	No of videos
Anger	2894	653
Contempt	3317	1385
Disgust	3061	828
Fear	3166	787
Happiness	3577	1488
Neutral	3491	1395
Sadness	3576	859
Surprise	3553	1330
Total	26635	6920

**Fig. 6:**
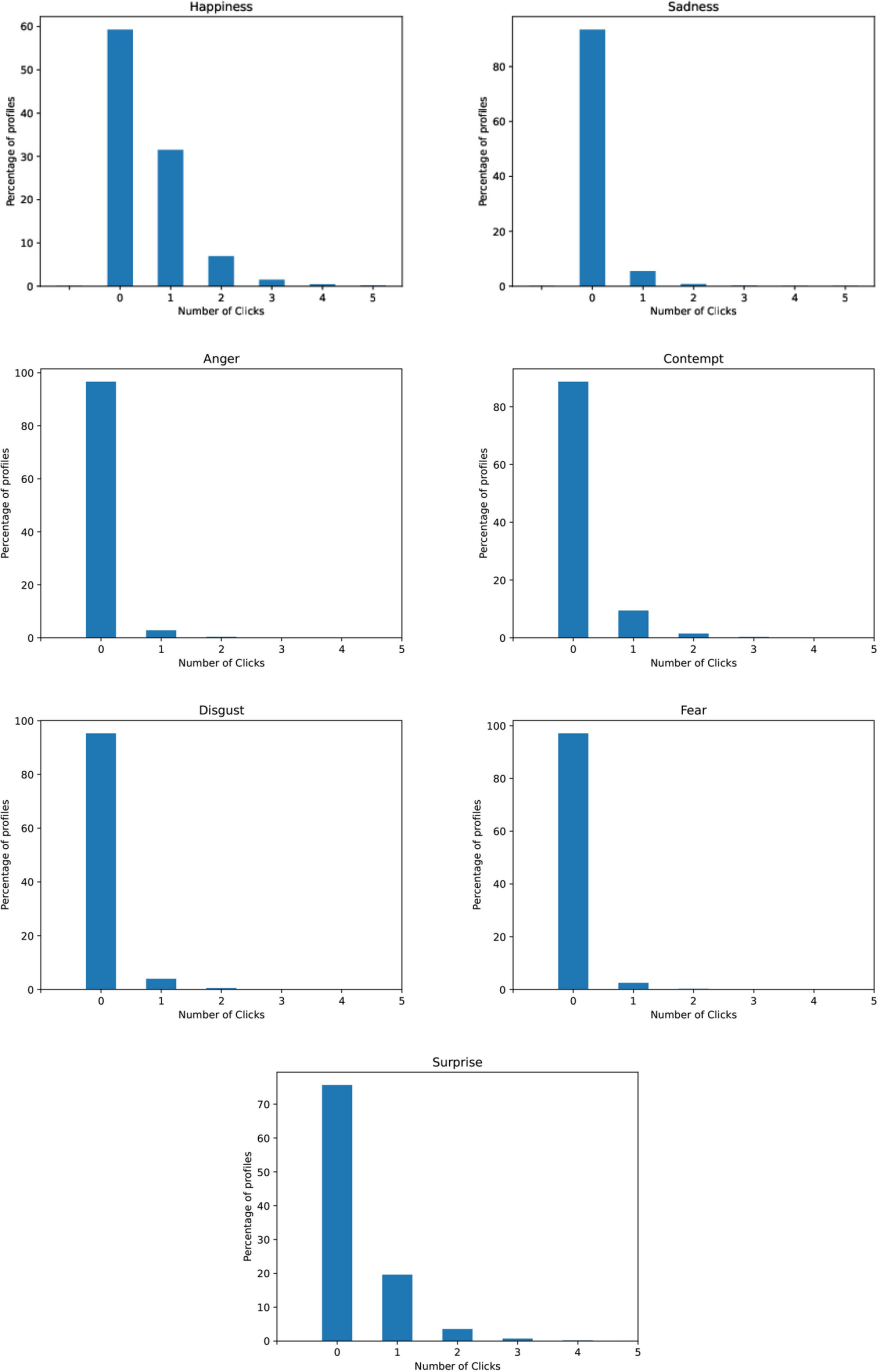
System1’s Test Your Ad data: distribution of number of emotion clicks per user per video, not adjusted for video duration.

**Table 3:** System1’s Test Your Ad data: Average accuracy of the video classifier broken down by input modality, number of video frames and pre-training method.

Modality	Frames	Pre-training	Accuracy
RGB	4	Imagenet	38.8
RGB+audio	4	Imagenet	42.4
RGB	8	Imagenet	38.9
**RGB+audio**	**8**	**Imagenet**	**43.2**
RGB	16	Imagenet	38.8
RGB+audio	16	Imagenet	42.2
RGB	4	INET21K	39.7
RGB+audio	4	INET21K	43.4
RGB	8	INET21K	41.2
RGB+audio	8	INET21K	43.5
RGB	16	INET21K	40.9
**RGB+audio**	**16**	**INET21K**	**43.6**

**Table 4:** System1’s Test Your Ad data: Balanced accuracy and test set size achieved by the video classifier broken down by emotion.

Emotion	Balanced Accuracy	Test Set Size (clips)
Anger	28.4	208
Contempt	42.3	269
Disgust	26.7	243
Fear	50.7	300
Happiness	55.8	326
Sadness	60.2	344
Surprise	51.1	325

**Fig. 7:**
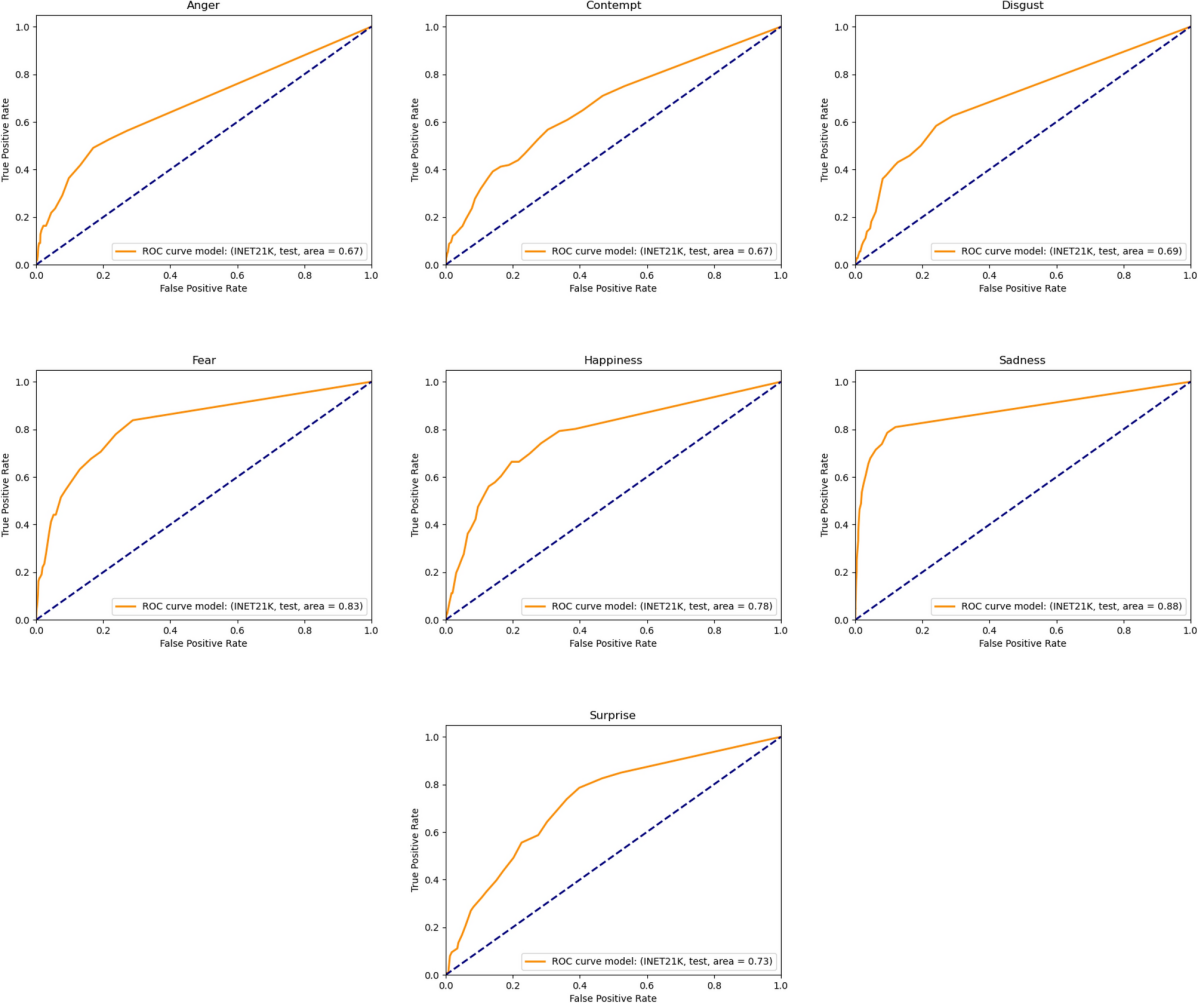
ROC curves for predicting the presence of emotion jumps in full-length video ads using our best CNN model (16 frames, RGB + audio input, pretrained on INET21K).

## Supplementary Information

Supplementary Information.

## Data Availability

To foster further research in video emotion recognition, we are providing non-commercial access to a curated subset of System1’s Test Your Ad dataset. This subset comprises 5-second video clips of publicly available advertisements that encapsulate the core viewer emotional responses, which were instrumental in generating the results presented in our paper. These clips are publicly available for research purposes to facilitate model training and evaluation. In addition to the dataset, we are also sharing the trained neural network weights and the complete Python codebase utilized for training and inference processes. We have established a public GitHub repository (https://github.com/gmontana/DecodingViewerEmotions) to host and distribute these resources, enabling seamless access and collaboration within the research community. Due to the substantial size of the dataset and trained model weights, which exceeds the limits of common dataset hosting platforms, these resources are hosted on servers at the University of Warwick. Interested researchers can directly request the download link by contacting the corresponding author at g.montana@warwick.ac.uk. It is important to note that the complete and original full-length video advertisements are publicly available and form part of the dataset which remains proprietary and under the custody of System1 Group PLC. However, this dataset may be accessible upon request (legal@system1group.com) for legitimate academic pursuits, subject to appropriate clearances and approvals. The advertisement snippets included in this publication are used solely for illustrative purposes to support academic research and analysis. These snippets are provided for criticism, review, and educational purposes only. All snippets are limited in scope and are not intended to replicate, replace, or serve as a substitute for the original content.
