# Supplementary material for: Decoding viewer emotions in video ads
Source: Sci Rep. 2024 Nov 2;14:26382. doi: 10.1038/s41598-024-76968-9 (PMC11530461; doi:10.1038/s41598-024-76968-9)
Supplement: Supplementary file 1 — Supplementary Information. [file 41598_2024_76968_MOESM1_ESM.pdf]

Decoding Viewer Emotions in Video Ads
Supplementary Material

Alexey Antonov[2], Shravan Sampath Kumar[4], Jiefei Wei[1], William Headley[4], Orlando Wood[4], and Giovanni Montana[1,2,3,*]

[1]Department of Statistics, University of Warwick
[2]WMG, University of Warwick
[3]Alan Turing Institute
[4]System1 Research
[*]g.montana@warwick.ac.uk

Rating	Videos	Anger	Contempt	Disgust	Fear	Happiness	Sadness	Surprise
1+	15,188	3.4%	6.9%	4.3%	3.8%	0.9%	3.3%	2.4%
2+	9,762	1.1%	2.8%	1.3%	1.6%	3.2%	1.0%	5.6%
3+	4,370	0.9%	1.5%	1.0%	1.3%	14.1%	0.6%	7.7%
4+	1,126	1.2%	1.1%	0.9%	1.5%	28.3%	0.7%	7.7%
5+	306	0.7%	0.0%	0.0%	3.6%	49.0%	0.0%	7.2%

Table S1: Distribution of video ads in the Test Your Ad dataset by star rating. The columns report the percentage of videos containing at least one emotion jump for each rating category (e.g. 48.8% of 5+ star ads had a Happiness jump, while less than 1% of 1+ star ads had a Happiness jump).

Percentile	Test Sample Size	Accuracy
3%	13,098	36.5
2%	8,801	39.1
1%	4,702	40.9
0.5%	2,387	43.6
0.1%	473	42.7

Table S2: Test Your Ad data: test set size and average classification accuracy when using different cutoff percentiles of the emotional jump distribution to define positive examples for model training.

Method	Type	Modality	Frames	PreTrained	Accuracy
TSM (2019, [5])	2D CNN	RGB	8	ImageNet	74.2
TSAM	2D CNN	RGB	8	ImageNet	73.3
TSAM	2D CNN	RGB+audio	8+1	ImageNet	75.8
TSAM	2D CNN	RGB	8	INet21K	75.8
TSAM	2D CNN	RGB+audio	8+1	INet21K	77.8
TSM (2019, [5])	2D CNN	RGB	16	ImageNet	74.7
TSAM	2D CNN	RGB	16	ImageNet	73.8
TSAM	2D CNN	RGB+audio	16+1	ImageNet	76.4
TSAM	2D CNN	RGB	16	INet21K	76.3
**TSAM**	**2D CNN**	**RGB+audio**	**16+1**	**INet21K**	**78.1**
X3D-M(2020, [2])	3D CNN	RGB	16	-	76.0
X3D-L(2020, [2])	3D CNN	RGB	16	-	78.2
ViViT-L(2021, [1])	Transformer	RGB	16	INet21K	80.6
UniFormer-S(2022, [4])	Transformer	RGB	16	ImageNet	80.8

Table S3: Comparison of TSAM performance on Kinetics-400 action recognition benchmark versus state-of-the-art 2D CNN models using RGB modality with 16 frames + 1 audio frame input. TSAM achieves state-of-the-art accuracy among 2D CNN architectures on this benchmark.

Method	Type	Frames	ShiftDepth	PreTrained	Accuracy
TSM (2019, [5])	2D CNN	8	1	ImageNet	45.6
TSM (2019, [5])	2D CNN	16	1	ImageNet	47.2
TSAM	2D CNN	16	1	INet21K	50.1
TSAM	2D CNN	16	2	INet21K	50.4
TSAM	2D CNN	16	3	INet21K	50.7
TSAM	2D CNN	16	4	INet21K	51.1
TSAM	2D CNN	16	5	INet21K	51.0
**TSAM**	**2D CNN**	**16**	**4**	**Kinetics400**	**52.1**
CT-Net(2021, [3])	3D CNN	16	-	ImageNet	52.5
UniFormer-S(2022,[4])	Transformer	16	-	Kinetics400	53.8

Table S4: Performance comparison of TSAM versus state-of-the-art 2D CNN models on the Something-Something V1 benchmark using 16 frame RGB input. TSAM achieves state-of-the-art accuracy among 2D CNN architectures.

References

[1] Anurag Arnab, Mostafa Dehghani, Georg Heigold, Chen Sun, Mario Lucic, and Cordelia Schmid. Vivit: A video vision transformer. *CoRR*, abs/2103.15691, 2021.

[2] Christoph Feichtenhofer. X3D: expanding architectures for efficient video recognition. *CoRR*, abs/2004.04730, 2020.

[3] Kunchang Li, Xianhang Li, Yali Wang, Jun Wang, and Yu Qiao. Ct-net: Channel tensorization network for video classification. *CoRR*, abs/2106.01603, 2021.

[4] Kunchang Li, Yali Wang, Gao Peng, Guanglu Song, Yu Liu, Hongsheng Li, and Yu Qiao. Uniformer: Unified transformer for efficient spatial-temporal representation learning. In *International Conference on Learning Representations*, 2022.

[5] Ji Lin, Chuang Gan, and Song Han. TSM: Temporal shift module for efficient video understanding. In *Proceedings of the IEEE International Conference on Computer Vision*, volume 2019-October, 2019.
